# Self-Harm and Suicide-Related Content on TikTok: Thematic Analysis

**DOI:** 10.2196/77828

**Published:** 2025-09-18

**Authors:** Gillian Grant-Allen, Lezhi Wang, Jasmine Amini, Simran Dhaliwal, Mark Sinyor, Rachel HB Mitchell

**Affiliations:** 1 Hurvitz Brain Sciences Program Evaluative Clinical Sciences Platform Sunnybrook Research Institute Toronto, ON Canada; 2 Temerty Faculty of Medicine University of Toronto Toronto, ON Canada; 3 Ontario Institute for Studies in Education University of Toronto Toronto, ON Canada; 4 Institute of Medical Science University of Toronto Toronto, ON Canada; 5 Department of Psychiatry Sunnybrook Health Science Centre Toronto, ON Canada; 6 Department of Psychiatry Temerty Faculty of Medicine University of Toronto Toronto, ON Canada

**Keywords:** self-injurious behavior, self-harm, suicide, social media, thematic analysis, TikTok, youth mental health, glamorization, content regulation, online safety, social contagion

## Abstract

**Background:**

Social media platforms, such as TikTok, may be powerful vectors for transmission of both harmful and helpful self-harm and suicide-related content; however, this has not been rigorously studied.

**Objective:**

This study aims to identify and understand the themes and overall characteristics of videos related to self-harm and suicide on TikTok.

**Methods:**

Snowball sampling was used to identify the 10 most-viewed TikTok hashtags related to self-harm and suicide, which were then used to select the most-viewed English-language posts up to June 2023. An inductive coding reliability approach to thematic analysis was iteratively applied by 2 independent coders to identify and analyze common themes within the videos.

**Results:**

In total, 188 videos were included in the thematic analysis. Five main themes and 2 subthemes were identified: emotional distress, hope and recovery-based messaging, grief and memorialization of those who died by suicide, social functions associated with self-harm and suicide-related content (subthemes: gallows humor and sarcasm; glamorization of self-harm and suicide-related behavior), and shame and guilt associated with self-harm and suicide-related behavior.

**Conclusions:**

Self-harm and suicide-related content on TikTok was diverse, encompassing both potentially harmful (eg, normalization of self-harm behavior) and helpful (eg, recovery-focused messaging) characteristics. Therefore, a multifaceted and collaborative approach is needed to address the risks of potentially harmful content while leveraging the positive characteristics to promote the safety and well-being of TikTok users.

## Introduction

### Background

Suicide and self-harm among youth are growing public health concerns. Suicide is the second leading cause of death among youth aged 24 years or younger, with suicide rates among youth rising relatively consistently over the past 2 decades [1-5]. Rates of self-harm among youth have also risen since 2008 in several industrialized countries, including the United States, Canada, Australia, and the United Kingdom [6-10]. The relationship between social media and youth mental health is multifaceted, with social media offering benefits, such as opportunities for social connection and self-expression, as well as posing challenges, including association with depressive symptoms and suicide-related behaviors among frequent users [11,12]. Importantly, social media is an effective medium for social contagion: the “spread of behaviours, attitudes, and affect” through “social aggregates from one member to another” [13]. Social contagion is a powerful vector for self-harm and suicide-related behaviors, particularly among youth, who are among the most susceptible to its effects [14]. Content depicting self-harm and suicide-related behaviors shared on social media may contribute to an increase in these behaviors among viewers via social contagion [15-17].

TikTok (ByteDance Ltd) is a public streaming social media platform for user-created short-form video content and is one of the fastest-growing social media apps among youth. It is widely assumed that young adolescents make up the largest share of TikTok’s global user base [18]. As of 2024, 93% of TikTok’s 1 billion users were aged between 13 and 34 years, and 57% of the teenagers in the United States reported using TikTok at least once per day [19]. TikTok hosts a vast amount of user-generated mental health–related content [20,21]. Although TikTok uses community guidelines that prohibit content “depicting, promoting, normalizing, or glorifying activities that could lead to suicide or self-harm,” including hashtags, such as #suicide or #self-harm, these restrictions are easily circumvented through the use of euphemistic references to self-harm and suicide or purposeful misspelling [22,23]. This terminology, known as “algospeak”, enables creators to share harmful or prohibited content without detection and removal by TikTok’s algorithm [24]. Therefore, the circumvention of community guidelines and consequent availability of suicide and self-harm–related content on TikTok may increase social contagion relating to these maladaptive behaviors. Despite this potential risk, TikTok users have reported benefits of the platform, including being a medium for creative expression, activism, and social connection to others with similar lived experiences [23,25].

### Objective

Given the ubiquity of TikTok use and risk of social contagion relating to suicide and self-harm behaviors, particularly among youth, it is essential to maintain awareness regarding the scope of self-harm and suicide-related social media content. This qualitative study aims to identify and characterize the multifaceted nature of self-harm and suicide-related content on TikTok.

## Methods

### Operationalization of Self-Harm

In this study, self-harm behaviors were conceptualized as a continuum rather than distinct intent-based categories. This decision was informed by several important considerations. First, while individuals might engage in self-harm behaviors with or without suicidal intent, there was no reliable way to infer intent solely from the context of a TikTok post, the only exception being if this intent was explicitly stated. Moreover, suicidal intent might be dynamic and, regardless of intent, previous studies suggested self-harm behaviors were longitudinally predictive of suicide [26,27]. Second, to accurately capture the nuances of self-harm behaviors while also avoiding potentially incorrect or arbitrary distinctions regarding intent, we defined self-harm as any “intentional self-poisoning or self-injury, irrespective of type of motive or the extent of suicidal intent” [28].

### Sampling Strategy

**Figure 1:**
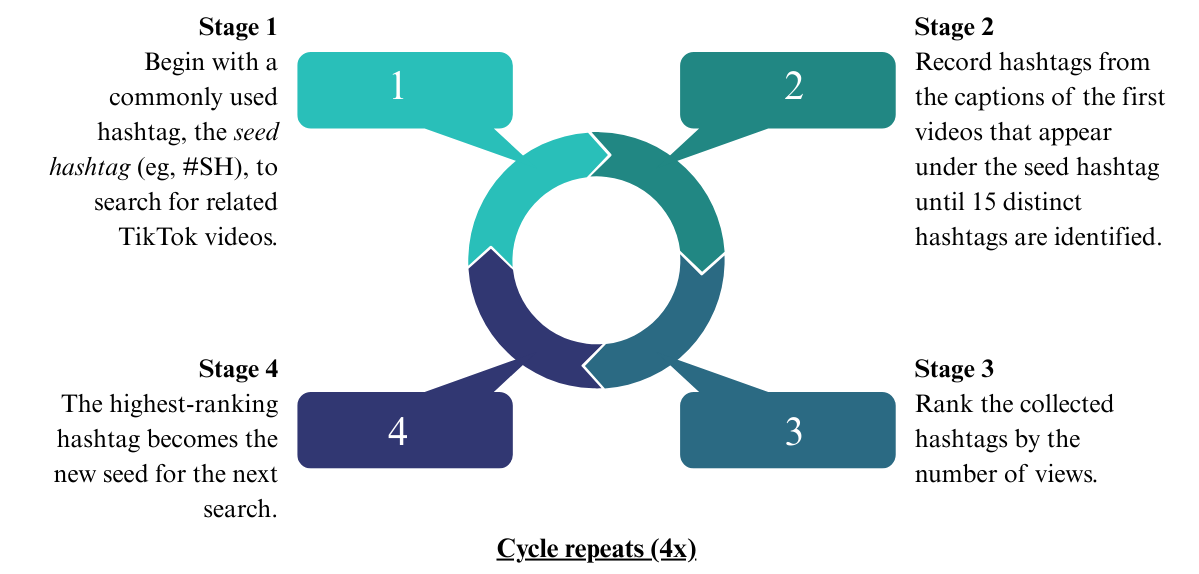
Snowball sampling process to identify TikTok hashtags related to self-harm and suicide.

A snowball sampling procedure was used to identify 10 of the most-viewed TikTok hashtags related to self-harm and suicide (Figure 1). Snowball sampling is an effective method for obtaining information on user-generated hashtags, given its dynamic and diachronic properties [29]. It is also particularly suited for capturing hashtags with euphemistic references to self-harm and suicide, which are common on TikTok [30-32].

For the purposes of this study, a new TikTok account was created to avoid algorithmic bias from previous viewing patterns. Similar to existing qualitative analyses of TikTok content, the videos appearing first under each hashtag were used to reflect a typical user’s experience [32]. Inclusion criteria for TikTok hashtags were a direct mention of suicide or self-harm (eg, #suicidepreventionmonth) or a euphemistic reference to suicide or self-harm. Examples included substituting characters using homoglyphs and acronyms or intentionally misspelling words related to self-harm or suicide (eg, #sh, #suiicide, #selfhaarm, or #s3lfcutting). Hashtags from non-English TikTok posts were excluded.

The hashtags that were searched to obtain TikTok videos were determined using an iterative snowball sampling process from June 13, 2023, to June 20, 2023. A single hashtag (#SH) was searched on the TikTok platform to yield the videos appearing first. The #SH hashtag, a common acronym for self-harm, was selected by reviewing gray literature. The first 15 hashtags in the captions of videos under the “#SH” search that met the inclusion criteria were ranked by view count published on the platform. The most viewed hashtag (#suicideprevention) was then selected, and the process was repeated; the video captions of the search results were used to identify the 15 hashtags appearing first that met the inclusion criteria. The hashtag list was then reranked by view count, and 2 additional searches were iteratively conducted using the next most viewed hashtags (#shawareness and #suicideawareness, respectively). A total of 4 searches were conducted to ensure data saturation (ie, no further unique hashtags meeting inclusion criteria emerged), and hashtags relating to both suicide and self-harm were searched equally. The final hashtag sample contained 35 unique hashtags ranked according to views. The 10 most-viewed hashtags were used to compile the sample of videos for analysis.

The top video posts under each hashtag search were downloaded to form the video sample. A target of 200 videos was selected based on convention from other thematic analyses of TikTok content [32-35]. Given the difference in view count for each of the searched hashtags, incrementally fewer videos were sampled from the lesser-viewed hashtags (Table 1 and Multimedia Appendix 1).

**Table 1:** TikTok hashtags with global view counts and number of videos sampled (N=200).

Hashtags	Views	Date and time searched	Videos sampled, n (%)
#sh^a^	7.4 billion	June 13, 2023; 8:45 AM	29 (14.5)
#suicideprevention^a^	1.3 billion	June 13, 2023; 2:32 PM	27 (13.5)
#suicideawareness^a^	876.8 million	June 15, 2023; 2:01 PM	25 (12.5)
#od	866.6 million	June 13, 2023; 2:32 PM	23 (11.5)
#suicicideawarnes	583.1 million	June 15, 2023; 2:01 PM	21 (10.5)
#suicidepreventionmonth	451.3 million	June 15, 2023; 2:01 PM	19 (9.5)
#shawareness^a^	318.3 million	June 14, 2023; 1:32 PM	17 (8.5)
#suicideawarness	303.7 million	June 15, 2023; 2:02 PM	15 (7.5)
#sewerslideawerness	131.2 million	June 13, 2023; 2:32 PM	13 (6.5)
#shrecovey	108.4 million	June 13, 2023; 2:32 PM	11 (5.5)

^a^Used as seed hashtags in the snowball sampling process.

### Coding and Thematic Analysis

**Figure 2:**
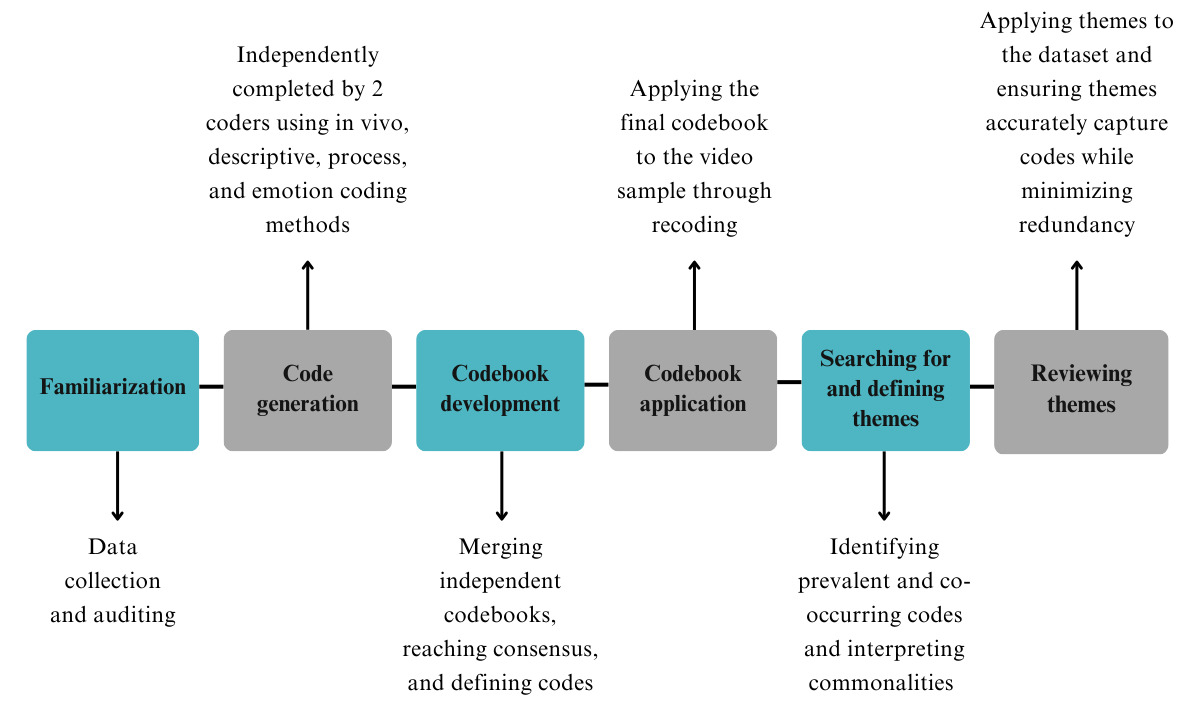
The thematic analysis process.

A 6-phase, inductive coding reliability approach to thematic analysis, as described by Clarke and Braun [36,37], was used to identify themes within the data (Figure 2). The Enhancing Transparency in Reporting the Synthesis of Qualitative Research (ENTREQ) guidelines were also applied [38].

Data familiarization was performed by 2 coders (GG-A and LW), who independently viewed all videos 3 or more times. Then, coding occurred in 2 iterative and recursive stages: initial coding and refined coding. During initial coding, coders independently used analytic memo writing and repeated viewings to note potential codes and themes. Each video was coded with several methods, including descriptive, in vivo, process, and emotion coding. Refined coding entailed discussions regarding nascent codes within the selected video content, ensuring each code was well defined.

Once the preliminary codebook was established, the coders independently coded all video posts in the sample (n=188 after duplicates were removed). A final codebook was generated (n=191 codes), defined, and used to recode the sample. Themes were subsequently generated by collating relevant codes and applying an inductive approach through face-to-face discussion between the primary coders and an additional moderator (JA). Relationships between codes and candidate themes were considered, and subthemes were generated, where applicable. Definitions for each theme were then created by collating relevant data extracts, accounting for possible overlap between themes and relevance of themes to the research question.

### Ethical Considerations

All data used in this study were publicly accessible TikTok videos, which did not require a user login to view. There was no direct interaction with users, and we did not access content marked as “private.” According to the Tri-Council Policy Statement Ethical Conduct for Research Involving Humans, Article 2.2, research does not require research ethics board review when it relies exclusively on information that is publicly available or in the public domain where individuals have no reasonable expectation of privacy [39]. As this study analyzed only publicly posted TikTok videos created and shared by users on a public platform, research ethics board approval was not required.

Based on the criteria proposed by Eysenbach and Till [40] for studying internet communities, informed consent was not obtained from creators. Specifically, informed consent was deemed unnecessary because the study consisted of passive analysis only (with no active involvement in the TikTok community by researchers) and all videos analyzed were publicly available with no reasonable expectation of privacy. Furthermore, because creators posting self-harm and suicide-related content on TikTok may be considered an at-risk population, confidentiality was maintained by withholding from the paper all identifying information (usernames, profile pictures, links to the videos) to protect creator anonymity and mitigate any potential harm from our results.

## Results

### Overview

**Figure 3:**
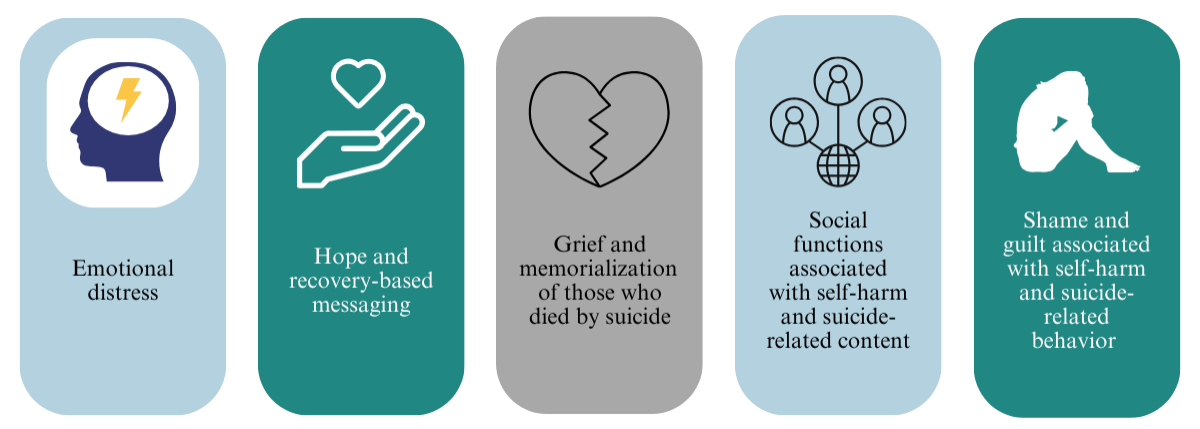
Key themes identified in self-harm and suicide-related content on TikTok.

The global views for the hashtags used in the snowball sampling process ranged from 7.4 billion for #sh to 108.4 million for #shrecovery (Table 1 and Multimedia Appendix 1). The thematic analysis identified five main themes (Figure 3) and two subthemes: (1) emotional distress, (2) hope and recovery-based messaging, (3) grief and memorialization of those who died by suicide, (4) social functions associated with self-harm and suicide-related content—gallows humor and sarcasm (subtheme 4.1) and glamorization of self-harm and suicide-related behavior (subtheme 4.2), and (5) shame and guilt associated with self-harm and suicide-related behavior.

### Theme 1: Emotional Distress

Videos in the emotional distress category were characterized by content that conveyed, either explicitly or implicitly, messages of hopelessness, loneliness, numbness, or frustration. The content had a negative tone and featured references to the creator’s personal self-harm and suicide-related behaviors.

Feelings of entrapment or being “stuck” in a cycle of suicidal ideation, self-harm, and suicide-related behaviors were common. Creators expressed frustration over the persistence of suicidal thoughts and self-harming behaviors, despite efforts to improve their mental health. This frustration was illustrated through adapted song lyrics, such as 1 video with music overlaid with the text “voices in your head screaming kys [kill yourself]…please understand I’m trying my hardest, my head’s a mess but I’m trying regardless.” This frustration was also articulated as a desire to “give up.” For example, 1 video featured a quote that read “I think I hit the point in life where I’m just done…I cried, I tried, but everything is crashing down…this time I’m to [*sic*] tired to fight back” while a melancholic video clip and song played in the background.

Ambivalence about living and dying by suicide was expressed. For example, suicide was referred to as a backup plan if the creator’s mental health did not improve. One video began with the text prompt “wyd [what you doing] if it don’t get better?” to which the creator replied via lip-synching song lyrics “pills and potions, we’re overdosing.”

### Theme 2: Hope and Recovery-Based Messaging

Videos categorized as containing hope and recovery-based messaging included content that discouraged self-harm and suicide-related behavior. Creators in this category referred to their lived experience and used one or both of the following approaches to dissuade audiences from engaging in suicide and self-harm behavior: (1) emotional appeals that conveyed messages of hope, validation, and recovery and (2) logical or practical appeals that emphasized deterrents, such as the physical health consequences or feelings of guilt associated with suicide-related or self-harm behaviors, including their impact on family and friends.

Videos conveying messages of hope typically included creators recounting their experiences of recovery from engaging in self-harm or suicide-related behavior. Creators often discussed milestones in their self-harm recovery journey, such as the duration of time they were able to abstain from self-harming or their acceptance of their self-harm scars. These videos ended with affirming messages, such as reminding viewers they were not alone and encouraging them to seek help if needed.

Other posts within this content category emphasized physical health consequences from suicide attempts, such as severe pain while recovering from an intentional medication overdose. Such posts also highlighted the aftermath of self-harm, such as “the cleanup, risk of infection, accidental unalive [ending your life]*.*” Many spoke candidly about the guilt and pain they felt due to the distress caused to their families. In addition to narrative-based content, a series of self-harm prevention and recovery videos within this theme offered concrete strategies for adaptive coping. For example, one creator used her self-harm scars to produce a piece of art, while another individual demonstrated strategies to resist self-harm urges.

### Theme 3: Grief and Memorialization of Those Who Died by Suicide

Grief and memorialization following suicide death formed a major thematic category. Content within this category emphasized either the personal experience of grief following the death of someone due to suicide or the memorialization of those who have died by suicide.

Videos conveying the complex process of grief following the death of a loved one by suicide varied substantially in tone and emotion. Some expressed sadness or emptiness, whereas others conveyed anger, frustration, or confusion. Trends included video montages of bereaved loved ones, typically either the creator or their children, emotionally expressing grief (eg, crying) over the suicide decedent. These videos were overlaid with text messages illustrating the pain of coping with this grief; 1 video example read “Suicide doesn’t take away the pain…it gives it to someone else*.*”

Content that focused on the memorialization of someone who died due to suicide had a nostalgic or neutral tone. These videos focused on the life and accomplishments of the individual who died by suicide rather than the circumstances of their death. Creators recounted special memories with their loved ones as well as their loved ones’ interests and positive personal characteristics. In a video, a creator described her partner, who died by suicide, writing, “[he] cared so deeply about everyone in his life…all he wanted was to take care of people*.*”

Implicit within most, if not all, grief and memorialization content was a desire to bring awareness to suicide. One trend included montages of photos or videos in the timeline leading up to a suicide attempt or suicide. These montages depicted the individual who died by or attempted suicide as happy, social, and engaged, countering societal perceptions of individuals with suicidal ideation as visibly despondent and withdrawn. A key explicit message in the video captions was that external appearances of social engagement and positive affect may not correlate with suicidal ideation or behavior.

### Theme 4: Social Functions Associated With Self-Harm and Suicide-Related Content

#### Overview

“Social function” refers to the ways in which self-harm and suicide-related behaviors influence relationships, contribute to group identification, and foster in-group construction. Content within this theme was divided into 2 subthemes: implicit and covert humor that included community symbols to describe experiences related to self-harm and suicidal ideation (subtheme 4.1) and glamorization (subtheme 4.2). Other videos discussed the social impacts of stigma relating to self-harm and suicide-related behaviors. These included examples such as real and imagined experiences of self-harm or suicide-related behavior disclosure to friends and family members and their associated feelings of isolation, fear, shame, and guilt.

#### Subtheme 4.1: Gallows Humor and Sarcasm

Content within this subtheme contained gallows humor and sarcasm to refer to self-harm behavior and suicidal ideation. For instance, 1 video featured an in-video heading of “reasons why I’m still alive :)” followed by a black screen with no listed reasons, implying a lack of perceived reasons to live. Another video showcased a user with the in-video caption “since it’s suicide prevention month here’s what I would have missed if I went through with it,” followed by an image of the user in a hospital and the in-video caption “getting cancer*.*”

#### Subtheme 4.2: Glamorization of Self-Harm and Suicide-Related Behavior

Content within this subtheme conveyed a tone of glamorization. Many videos included implicit references to self-harm and self-harm scars, such as the use of a cutting board as a symbol for self-harm, covert references to self-harm tracking apps, or tattoo designs resembling self-harm scars. Other videos contained descriptions of feeling inadequate or fearing that self-harm scars are fading, rendering one’s experience of self-harm “invalid.” For example, 1 post showed a user’s wrist with the in-video caption “People showing their scars with this filter,” followed by “It’s kind of triggering now I feel like mine aren’t ‘bad enough’ because they don’t show up*.*” Another post featured a video of a carrot sharpener with a large blade, accompanied by the audio “it’s so cute! Capitalism really popped off today,” which implicitly alluded to the desire to self-harm, along with comments such as “can I have it? You can keep the red part,” and “I-uhm…can I…borrow…th-that?” In another post, the in-video caption read “an object I can relate to but I won’t say why,” followed by images of a cutting board and a winky face.

### Theme 5: Shame and Guilt Associated With Self-Harm and Suicide-Related Behavior

Videos under this theme highlighted the vulnerability and social impacts of self-harm and suicide-related behavior, including resultant feelings of guilt, shame, and self-consciousness about others seeing one’s self-harm scars. One creator shared, “It’s not worth it to try. Every day I have to walk out of my door of my house, and turn around and see my parents’ expression on their face like they’re never going to see me again, and it burns a hole right through me.” Another creator noted, “[I] can’t describe the amount of guilt and shame I felt knowing I almost put my friends and family through that [his suicide attempt].” Creators expressed that these feelings of guilt were a deterrent to disclosing self-harm or suicidal behaviors to loved ones and attempting suicide.

Creators also highlighted the fear associated with unwanted disclosure of self-harm and suicide-related behavior. In addition to fear, expressions of shame and guilt revolved around the desire to conceal or erase self-harm scars. Creators expressed sentiments, such as, “I wish I had no scars on my thighs like most people,” or “POV: They’re [scars are] white now so you can’t hide them*.*” In another video, a creator wrote, “When you have to constantly wear an arm sleeve so you’re not a walking trigger*.*”

## Discussion

### Principal Findings

This study examined a sample of self-harm and suicide-related content videos on TikTok. Through a search of 10 relevant hashtags, 188 TikTok posts were analyzed, and five themes were identified: (1) emotional distress, (2) hope and recovery-based messaging, (3) grief and memorialization of those who died by suicide, (4) social functions associated with self-harm and suicide-related content, and (5) shame and guilt associated with self-harm and suicide-related behavior. Most of the posts centered on personal experience with self-harm and suicide. Videos demonstrated multiple themes, and there was variability in how self-harm and suicide were portrayed, with both potentially harmful and protective content.

Several videos depicted potentially harmful characteristics, including extensive discussions of self-harm and suicide-related behaviors. These videos included details regarding personal precipitants of self-harm or suicide or graphic and specific details, such as the location on the body or methods. Another concerning, though less common, content category featured users posting while in acute emotional distress and openly expressing suicidal or self-harm ideation. At the most severe end, 2 posts from the sample depicted the creator actively engaging in self-harm or suicide-related behaviors. Such content deviates from the TikTok-specific community guidelines, general guidelines for media reporting on self-harm and suicide, and the more recent #chatsafe framework designed specifically for youth creating and consuming self-harm-related content online [41]. These guidelines caution against detailed descriptions of self-harm acts, even if users embed these details within an overall positive or hopeful narrative. The presence of TikTok content within our sample that deviated from these guidelines is concerning, especially given that such content is typically made by and for youth likely experiencing mental health difficulties. Together, this highlights the need for enhanced content moderation and educational efforts to mitigate the risks of all content on social media that may normalize and lead to social contagion of self-harm behaviors.

We identified communities on TikTok where users frequently made social connections through their experiences with self-harm and suicide-related behaviors through posting memes, inside jokes, and distinctive identifiers (eg, image of a barcode and cutting board) alluding to self-harm. Members of these communities could knowingly or unknowingly be exposing themselves to content that could potentially normalize their self-harm and suicide-related behavior. For creators of this content, this behavior may be further reinforced if validation is derived from social media metrics (eg, likes and comments). An in-group identity connected to self-harm may be harmful for several reasons. As youth engage in these communities, self-harm is not only reinforced but may become increasingly integral to their self-identity. In-group dynamics may prove challenging for clinical interventions, as individuals may have increased difficulty detaching from behaviors that have been normalized within their social context (eg, difficulty seeing self-harm scars fade). Furthermore, essential aspects of youth development, such as role exploration, identity formation, and the development of a subjective sense of belonging, are significantly shaped by in-group identities [42-45]. Given that youth are inherently more susceptible to the influence of such communities, there is a need for targeted interventions, such as educational initiatives, to cultivate online environments that are safer for youth while still promoting their sense of belonging [42,44].

In our sample, there were several montages memorializing celebrities and well-known social media personalities who died by suicide. Such content used stigmatizing language (ie, “committed suicide”) and depicted decedents as successful, happy, and energized. This portrayal appeared to be an attempt to challenge societal stereotypes about what a person contemplating suicide may look like. The existing literature indicates that memorializing celebrities who die by suicide increases the risk of imitative suicide-related behavior [46,47]. Celebrities and public figures are often perceived as role models; by extension, their acts, including suicide or suicidal behaviors, may be emulated by at-risk individuals [41]. Most existing literature in this domain explores suicide rate increases following celebrity death; less is currently understood about the influence of suicides by social media content creators.

There were many positive aspects of the self-harm and suicide-related content on TikTok identified in our analysis, including recovery-oriented material and the protective aspects of online self-harm and suicide-related communities. Under suicide and self-harm “awareness” and “prevention” hashtags, users shared their mental health journeys and conveyed messages of hope. Furthermore, the comment sections of these videos served as a space for social connection. Despite the potential for social contagion of self-harm and suicide within TikTok communities, these spaces also enable users to seek companionship, support, and validation. This may be particularly protective for youth who lack such support offline or face stigmatizing or marginalizing attitudes in response to their self-harm or suicide ideation and behaviors [47]. The relative anonymity afforded by TikTok appeared to encourage youth to disclose mental health challenges candidly. This openness may represent a valuable opportunity for researchers, clinicians, and educators to devise interventions aimed at supporting youth.

Youth interest in and the use of social media continues to grow. Stakeholders must attend to social media’s potentially harmful influence on self-harm and suicide-related behaviors while leveraging its popularity to promote youth mental health. In the context of TikTok, there are several strategies, both within and outside the app, which may concurrently support youth mental health. Within the app, enhancing content moderation that includes but is not limited to “algospeak” may mitigate the dissemination of harmful material on the platform, which is particularly important for at-risk youth. There is concern that some may perceive this measure as “censorship,” which may lead to increased self-stigma, distrust of suicide prevention efforts, or inadvertent migration of users to less regulated online environments, in the context of the rising rates of self-harm and suicide among youth. In 2024, TikTok revised its community guidelines to add a “Mental and Behavioral Health” section which states that the platform “does not allow showing, promoting, or sharing plans for suicide or self-harm” while permitting recovery-oriented stories, suicide and self-harm prevention content, and “accurate information that is trying to reduce panic about suicide hoaxes” [48]. However, it is at the very least crucial to have enforcement of the current community guidelines and transparent communication regarding content moderation so that users can be empowered to regulate their own content.

TikTok may also consider collaborating with external partners to deliver support to struggling users. As noted, the anonymity afforded by social media enables open discussions of mental health, which may be co-opted to connect youth to professional mental health support. This may include delivering mental health services via social media platforms through purpose-built apps or existing private groups and showing geographically appropriate mental health resources to users on their “For You” page. Recent scoping and systematic reviews examining the feasibility and effectiveness of social media–based interventions for youth mental health provide preliminary evidence of their ability to improve mental health outcomes, although controlled trials are needed [49-51]. In cases where real-time crisis support is inaccessible, asynchronous supports, such as safety planning toolkits, may also be valuable.

### Limitations

There are several limitations to this study. Although we used a rigorous search strategy, this study examined a small proportion of all available content related to self-harm and suicide on the TikTok app. As the videos appearing first under 10 of the most-viewed self-harm and suicide-related hashtags globally were used for the analysis, we may not have fully captured the nuance and scope of suicide- and self-harm–related content on TikTok. Furthermore, to avoid potentially incorrect or arbitrary distinctions regarding suicidal intent and appreciate that self-harm and suicide-related behaviors exist on a continuum, we did not analyze self-harm and suicide-related content as distinct entities. By doing so, we may have inadvertently missed details that apply to one phenomenon but not the other (eg, to attempt suicide but not self-harm or vice-versa). That being said, we made an explicit effort throughout the analysis to avoid conflating these distinct behaviors. The distinct but related nature of these behaviors is indeed demonstrated in our 5 themes, as there was only one that pertained exclusively to suicide (eg, theme 3) and one that pertained predominantly (but not exclusively) to self-harm (eg, theme 4). The other 3 themes represented both self-harm content and suicide-related content in roughly equal proportions, and most of the videos contained content that could not be reliably categorized as self-harm content or suicide-related content (eg, someone revealing their scars on their arm and talking about passive suicidal ideation). To gather videos for our sample, we created a new TikTok account; it is likely that the location of the devices used to access content (Toronto, Ontario) influenced the content that appeared in searches. TikTok’s For You feed uses user interactions, content information, and user information (which includes location, among other factors, such as time zone and day, device settings, and device type) to deliver personalized content to the user [52]. Consequently, our sample may overrepresent content popular in Toronto (Canada) and in North America more broadly while possibly underrepresenting content from other global regions. In addition, our search captured a specific time point, and it is possible that trends in self-harm and suicide-related content creation may have shifted since obtaining the video sample. The exclusion of non-English content further limited our search and the generalizability of our findings. Finally, as this is a TikTok-based study, we cannot comment on how suicide or self-harm is discussed on other social media platforms that are popular among youth.

### Conclusions

Youth are readily consuming and creating self-harm and suicide-related content on TikTok. Our results highlight the significant heterogeneity of this content. A considerable proportion of videos featured personal expressions of shame, distress, and grief associated with self-harm and suicide. Some content glamourized or normalized self-harm and suicide-related behavior, while other videos featured messages of hope and recovery. It is critical to devise a response that recognizes the nuance and complexity within the relationship between engaging with self-harm or suicide-related content on social media and engaging in these behaviors. A multifaceted approach that improves content regulation with concurrent transparent communication, includes educational initiatives, and provides support to struggling users may be a positive solution that validates youth experience while still prioritizing safety.

## Appendix

Multimedia Appendix 1 TikTok hashtags by global view count.

## Abbreviations

ENTREQ: Enhancing Transparency in Reporting the Synthesis of Qualitative Research

## Data Availability

The codebook and datasets generated or analyzed during this study are available from the corresponding author on reasonable request. The sample videos analyzed during this study are not publicly available to protect the privacy of the creators but are available from the corresponding author on reasonable request.

## References

[1] Hua LL, Lee J, Rahmandar MH, Sigel EJ, Committee on Adolescence, Council on Injury‚ Violence‚ And Poison Prevention (2024). Suicide and suicide risk in adolescents. Pediatrics.

[2] Moran P, Chandler A, Dudgeon P, Kirtley OJ, Knipe D, Pirkis J, Sinyor M, Allister R, Ansloos J, Ball MA, Chan LF, Darwin L, Derry KL, Hawton K, Heney V, Hetrick S, Li A, Machado DB, McAllister E, McDaid D, Mehra I, Niederkrotenthaler T, Nock MK, O'Keefe VM, Oquendo MA, Osafo J, Patel V, Pathare S, Peltier S, Roberts T, Robinson J, Shand F, Stirling F, Stoor JP, Swingler N, Turecki G, Venkatesh S, Waitoki W, Wright M, Yip PS, Spoelma MJ, Kapur N, O'Connor RC, Christensen H (2024). The Lancet Commission on self-harm. Lancet.

[3] Statistics Canada, Canadian Vital Statistics - death database (CVSD). Government of Canada.

[4] Suicide in Canada: key statistics (infographic). Canadian Institute of Health Information (CIHI).

[5] Curtin SC, Garnett MF Suicide and homicide death rates among youth and young adults aged 10–24: United States, 2001–2021. Centers for Disease Control and Prevention (CDC).

[6] Mercado MC, Holland K, Leemis RW, Stone DM, Wang J (2017). Trends in emergency department visits for nonfatal self-inflicted injuries among youth aged 10 to 24 years in the United States, 2001-2015. JAMA.

[7] Gardner W, Pajer K, Cloutier P, Zemek R, Currie L, Hatcher S, Colman I, Bell D, Gray C, Cappelli M, Duque DR, Lima I (2019). Changing rates of self-harm and mental disorders by sex in youths presenting to Ontario emergency departments: repeated cross-sectional study. Can J Psychiatry.

[8] Mitchell RHB, Toulany A, Chung H, Cohen E, Fu L, Strauss R, Vigod SN, Stukel TA, Moran K, Guttmann A, Kurdyak P, Artani A, Kopec M, Saunders NR (2023). Self-harm among youth during the first 28 months of the COVID-19 pandemic in Ontario, Canada: a population-based study. CMAJ.

[9] Mitchell RJ, Seah R, Ting HP, Curtis K, Foster K (2018). Intentional self-harm and assault hospitalisations and treatment cost of children in Australia over a 10-year period. Aust N Z J Public Health.

[10] Cybulski L, Ashcroft DM, Carr MJ, Garg S, Chew-Graham CA, Kapur N, Webb RT (2021). Temporal trends in annual incidence rates for psychiatric disorders and self-harm among children and adolescents in the UK, 2003-2018. BMC Psychiatry.

[11] Shannon H, Bush K, Villeneuve PJ, Hellemans KG, Guimond S (2022). Problematic social media use in adolescents and young adults: systematic review and meta-analysis. JMIR Ment Health.

[12] Macrynikola N, Auad E, Menjivar J, Miranda R (2021). Does social media use confer suicide risk? A systematic review of the evidence. Comput Human Behav Rep.

[13] Social contagion. American Psychological Association.

[14] Martínez V, Jiménez-Molina Á, Gerber MM (2023). Social contagion, violence, and suicide among adolescents. Curr Opin Psychiatry.

[15] Shoib S, Chandradasa M, Nahidi M, Amanda TW, Khan S, Saeed F, Swed S, Mazza M, Di Nicola M, Martinotti G, Di Giannantonio M, Armiya'u AY, De Berardis D (2022). Facebook and suicidal behaviour: user experiences of suicide notes, live-streaming, grieving and preventive strategies-a scoping review. Int J Environ Res Public Health.

[16] Emma Hilton C (2017). Unveiling self-harm behaviour: what can social media site Twitter tell us about self-harm? A qualitative exploration. J Clin Nurs.

[17] Brown RC, Fischer T, Goldwich DA, Plener PL (2020). "I just finally wanted to belong somewhere"-qualitative analysis of experiences with posting pictures of self-injury on Instagram. Front Psychiatry.

[18] McCashin D, Murphy CM (2023). Using TikTok for public and youth mental health - a systematic review and content analysis. Clin Child Psychol Psychiatry.

[19] Bestvater S How U.S. adults use TikTok. Pew Research Center.

[20] Pretorius C, McCashin D, Coyle D (2022). Mental health professionals as influencers on TikTok and Instagram: what role do they play in mental health literacy and help-seeking?. Internet Interv.

[21] (2024). TikTok community guideline: safety and civility. TikTok.

[22] TikTok community guideline: suicide and self-harm. TikTok.

[23] Lookingbill V, Le K (2024). “There’s always a way to get around the guidelines”: nonsuicidal self-injury and content moderation on TikTok. Soc Media Soc.

[24] Delkic M Leg booty? Panoramic? Seggs? How TikTok is changing language. The New York Times.

[25] Literat I, Kligler-Vilenchik N (2023). TikTok as a key platform for youth political expression: reflecting on the opportunities and stakes involved. Soc Media Soc.

[26] Boxer P (2010). Variations in risk and treatment factors among adolescents engaging in different types of deliberate self-harm in an inpatient sample. J Clin Child Adolesc Psychol.

[27] Klonsky ED (2011). Non-suicidal self-injury in United States adults: prevalence, sociodemographics, topography and functions. Psychol Med.

[28] Hawton K, Saunders KE, O'Connor RC (2012). Self-harm and suicide in adolescents. Lancet.

[29] Noy C (2008). Sampling knowledge: the hermeneutics of snowball sampling in qualitative research. Int J Soc Res Methodol.

[30] Sun T, Lim CC, Chung J, Cheng B, Davidson L, Tisdale C, Leung J, Gartner CE, Connor J, Hall WD, Chan GC (2023). Vaping on TikTok: a systematic thematic analysis. Tob Control.

[31] Rutherford BN, Sun T, Johnson B, Co S, Lim TL, Lim CC, Chiu V, Leung J, Stjepanovic D, Connor JP, Chan GC (2022). Getting high for likes: exploring cannabis-related content on TikTok. Drug Alcohol Rev.

[32] Herrick SS, Hallward L, Duncan LR (2021). "This is just how I cope": an inductive thematic analysis of eating disorder recovery content created and shared on TikTok using #EDrecovery. Int J Eat Disord.

[33] Foster M, Frith H, John M (2024). 'I'm still su!c!dal when you're done with the paperwork': an inductive framework thematic analysis of #camhs on TikTok. J Child Psychol Psychiatry.

[34] Davis HA, Kells MR, Roske C, Holzman S, Wildes JE (2023). A reflexive thematic analysis of #WhatIEatInADay on TikTok. Eat Behav.

[35] Ming S, Han J, Li M, Liu Y, Xie K, Lei B (2022). TikTok and adolescent vision health: content and information quality assessment of the top short videos related to myopia. Front Public Health.

[36] Clarke V, Braun V (2018). Using thematic analysis in counselling and psychotherapy research: a critical reflection. Couns Psychother Res.

[37] Braun V, Clarke V (2020). Can I use TA? Should I use TA? Should I not use TA? Comparing reflexive thematic analysis and other pattern-based qualitative analytic approaches. Couns Psychother Res.

[38] Tong A, Flemming K, McInnes E, Oliver S, Craig J (2012). Enhancing transparency in reporting the synthesis of qualitative research: ENTREQ. BMC Med Res Methodol.

[39] Tri-­Council policy statement: ethical conduct for research involving humans. Canadian Institutes of Health Research.

[40] Eysenbach G, Till JE (2001). Ethical issues in qualitative research on internet communities. BMJ.

[41] Robinson J, Hill NT, Thorn P, Battersby R, Teh Z, Reavley NJ, Pirkis J, Lamblin M, Rice S, Skehan J (2018). The #chatsafe project. Developing guidelines to help young people communicate safely about suicide on social media: A Delphi study. PLoS One.

[42] Young R, Sproeber N, Groschwitz RC, Preiss M, Plener PL (2014). Why alternative teenagers self-harm: exploring the link between non-suicidal self-injury, attempted suicide and adolescent identity. BMC Psychiatry.

[43] Bornholt LJ (2000). Social and personal aspects of self knowledge: a balance of individuality and belonging. Learn Instr.

[44] Brown BB, Lohr MJ (1987). Peer-group affiliation and adolescent self-esteem: an integration of ego-identity and symbolic-interaction theories. J Pers Soc Psychol.

[45] Turner JC, Oakes PJ (2011). The significance of the social identity concept for social psychology with reference to individualism, interactionism and social influence. Br J Soc Psychol.

[46] Sinyor M, Williams M, Niederkrotenthaler T (2019). Media depictions of possible suicide contagion among celebrities: a cause for concern and potential opportunities for prevention. Aust N Z J Psychiatry.

[47] Niederkrotenthaler T, Fu K, Yip PS, Fong DY, Stack S, Cheng Q, Pirkis J (2012). Changes in suicide rates following media reports on celebrity suicide: a meta-analysis. J Epidemiol Community Health.

[48] TikTok community guideline: mental and behavioral health. TikTok.

[49] Susi K, Glover-Ford F, Stewart A, Knowles Bevis R, Hawton K (2023). Research review: viewing self-harm images on the internet and social media platforms: systematic review of the impact and associated psychological mechanisms. J Child Psychol Psychiatry.

[50] Balt E, Mérelle S, Robinson J, Popma A, Creemers D, van den Brand I, van Bergen D, Rasing S, Mulder W, Gilissen R (2023). Social media use of adolescents who died by suicide: lessons from a psychological autopsy study. Child Adolesc Psychiatry Ment Health.

[51] Lavis A, Winter R (2020). #Online harms or benefits? An ethnographic analysis of the positives and negatives of peer-support around self-harm on social media. J Child Psychol Psychiatry.

[52] (2021). How TikTok recommends content. TikTok Support.

