# Supplementary figures and images for: Self-Harm and Suicide-Related Content on TikTok: Thematic Analysis
Source: J Med Internet Res. 2025 Sep 18;27:e77828. doi: 10.2196/77828 (PMC12491888; doi:10.2196/77828)

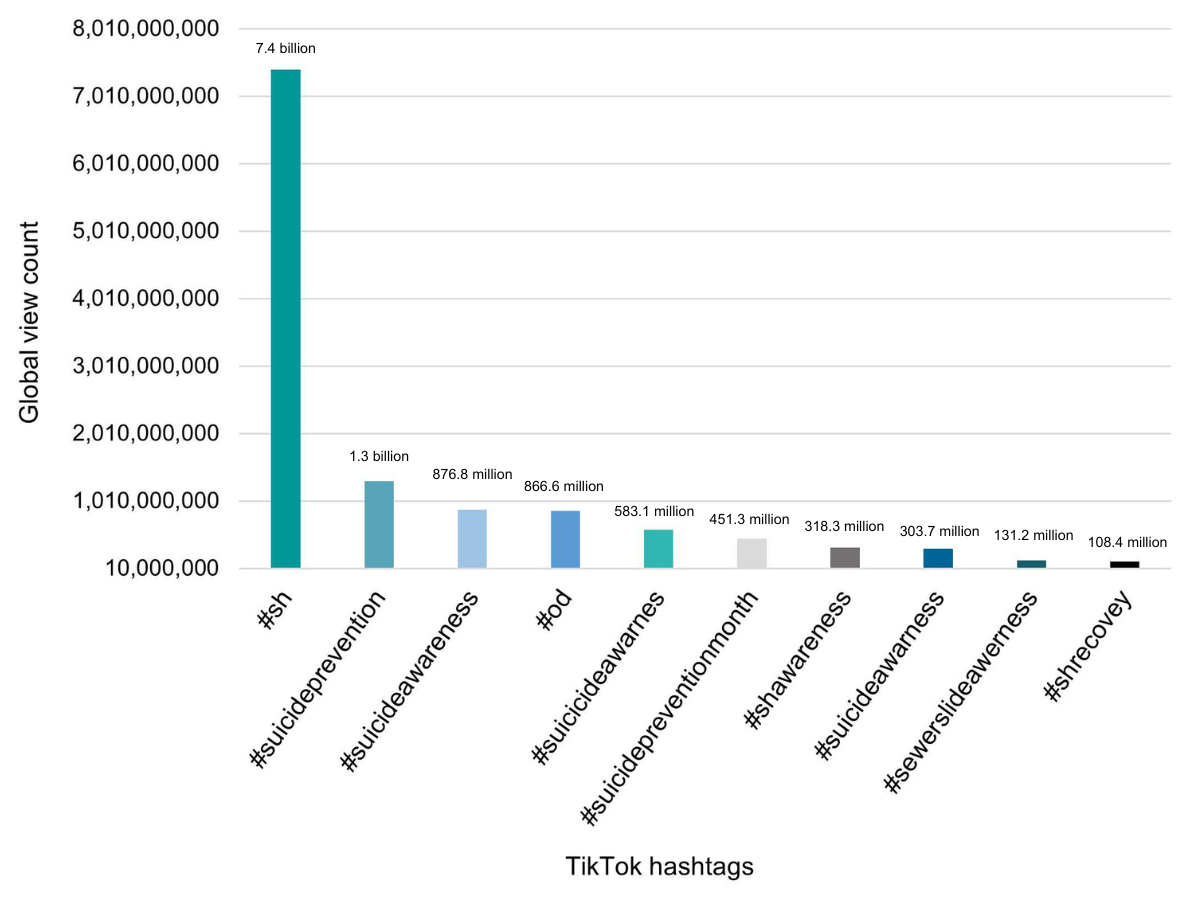

Supplement: Multimedia Appendix 1 [file jmir_v27i1e77828_app1.png]
